# A multicenter study on the application of artificial intelligence radiological characteristics to predict prognosis after percutaneous nephrolithotomy

**DOI:** 10.3389/fendo.2023.1184608

**Published:** 2023-09-15

**Authors:** Jian Hou, Xiangyang Wen, Genyi Qu, Wenwen Chen, Xiang Xu, Guoqing Wu, Ruidong Ji, Genggeng Wei, Tuo Liang, Wenyan Huang, Lin Xiong

**Affiliations:** ^1^ Division of Urology, Department of Surgery, The University of Hongkong-Shenzhen Hosipital, ShenZhen, China; ^2^ Department of Urology, Zhuzhou Central Hospital, Zhuzhou, China; ^3^ Department of Radiology, Zixing First People’s Hospital, Chenzhou, China

**Keywords:** artificial intelligence, clinical-radionics model, decision support system, renal staghorn stones, percutaneous nephrolithotomy

## Abstract

**Background:**

A model to predict preoperative outcomes after percutaneous nephrolithotomy (PCNL) with renal staghorn stones is developed to be an essential preoperative consultation tool.

**Objective:**

In this study, we constructed a predictive model for one-time stone clearance after PCNL for renal staghorn calculi, so as to predict the stone clearance rate of patients in one operation, and provide a reference direction for patients and clinicians.

**Methods:**

According to the 175 patients with renal staghorn stones undergoing PCNL at two centers, preoperative/postoperative variables were collected. After identifying characteristic variables using PCA analysis to avoid overfitting. A predictive model was developed for preoperative outcomes after PCNL in patients with renal staghorn stones. In addition, we repeatedly cross-validated their model’s predictive efficacy and clinical application using data from two different centers.

**Results:**

The study included 175 patients from two centers treated with PCNL. We used a training set and an external validation set. Radionics characteristics, deep migration learning, clinical characteristics, and DTL+Rad-signature were successfully constructed using machine learning based on patients’ pre/postoperative imaging characteristics and clinical variables using minimum absolute shrinkage and selection operator algorithms. In this study, DTL-Rad signal was found to be the outstanding predictor of stone clearance in patients with renal deer antler-like stones treated by PCNL. The DTL+Rad signature showed good discriminatory ability in both the training and external validation groups with AUC values of 0.871 (95% CI, 0.800-0.942) and 0.744 (95% CI, 0.617-0.871). The decision curve demonstrated the radiographic model’s clinical utility and illustrated specificities of 0.935 and 0.806, respectively.

**Conclusion:**

We found a prediction model combining imaging characteristics, neural networks, and clinical characteristics can be used as an effective preoperative prediction method.

## Introduction

**Figure 1:**
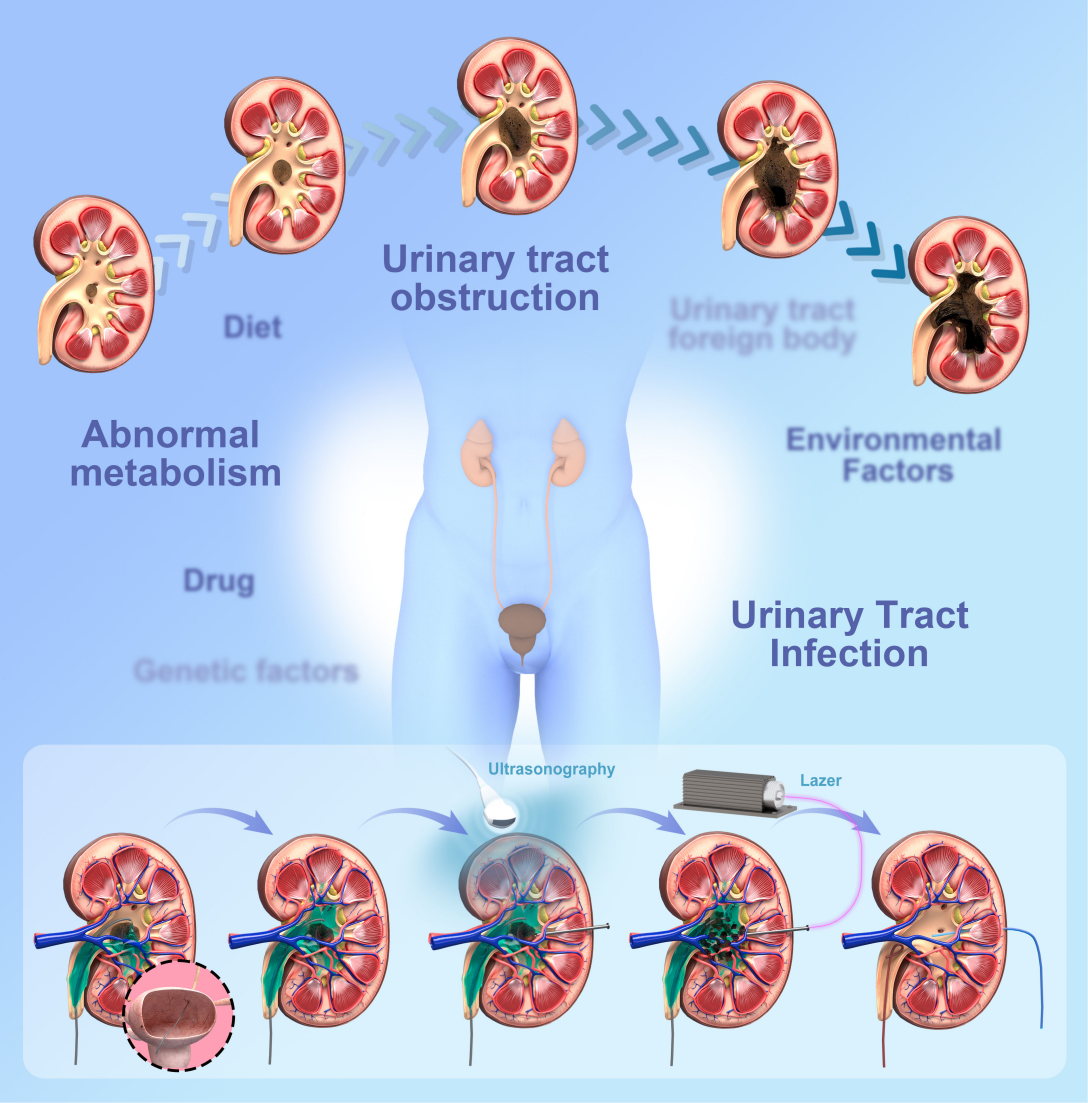
Diagram of kidney cast stone formation and surgery.

As a widespread urological disorder, the incidence of kidney stones varies from 1% to 20%. The prevalence of kidney stones is exceptionally high in western countries (>10%). Kidney stones are a widespread disease, affecting 5% of the US population (1), of which 10% to 20% are staghorn stones (2). Staghorn calculus includes both intact and partial integrity. Intact staghorn stones occupy more than 80% of the renal pelvis and collecting system, while partial stones occupy the renal pelvis and at least two calyces (3). Invasive procedures to treat this disease include percutaneous nephrolithotomy (PCNL) and retrograde intrarenal surgery (RIRS). PCNL has been the widely accepted method for staghorn stones (4), and the number of PCNL for staghorn stones has been significantly expanded for many years. Despite improvements in PCNL technical equipment, the high rate of perioperative complications and stone recurrence in PCNL remains a challenge for urologists (5, 6).(**Figure 1**)

At the same time, the Clinical Research Department of Sponge Urology Association (CROES) showed that the expected stoneless rate of staghorn calculi patients treated with PCNL was 56.9%. In comparison, the stoneless rate of non-staghorn calculi patients was 82.5% (7). Although several studies have demonstrated that the postoperative stoneless rate of PCNL is much better than various other procedures, there are still some cases of incomplete stone removal. Therefore, reducing the incidence of residual stone fragments after PCNL operation is essential to reducing patients’ financial stress and improving their quality of life.

Current research studied several leading scoring systems (STONE renal stone measurement, Geiger stone score-GSS and CROES nomination chart). In terms of predicting the results of patients with staghorn calculi after PCNL, their results confirmed that STONE nephroscopy was the only predictor of staghorn calculi without stones after PCNL (8, 9). In addition, their results indicated that stone-burden was strongly correlated with postoperative stoneless rate (10). Some mainstream analysis methods, i.e. GSS, STONE, CROES nomination map, and kidney stone test, have been proposed to provide a simple method for kidney stones. They all have two sides, and studies have shown that they are not comparable in the capabilities to calculate stoneless rates. Those limited prediction models of single-order parameters still do not achieve satisfactory results. Therefore, developing models with higher predictive power is essential to rapidly provide better guidance for treating renal deerstalker stones and optimize the outcomes of patients with renal deerstalker stones (11–13).

The latest development in computer auxiliary imaging technology makes it possible to conduct quantitative analysis from digital images in a high-throughput manner. In fact, this new approach, called radiology, has been shown to influence diagnostic and therapeutic strategies in oncology (14, 15). Additionally, some studies have shown that models based on radiology and neural network have the ability to predict the postoperative effects of specific surgical treatments (PCNL or SWL) (16, 17). This study performed a comparative analysis of several predictive models constructed by artificial intelligence deep transfer learning related to the postoperative prognosis of deer antler stones. Parameters of three recent mainstream models and radiologically relevant parameters were incorporated to screen for the best prognostic factors. The aim is to provide better guidance in developing treatment plans for renal deerstalker stones and to achieve optimal care for patients with renal deerstalker stones for better prediction.

**Figure 2:**
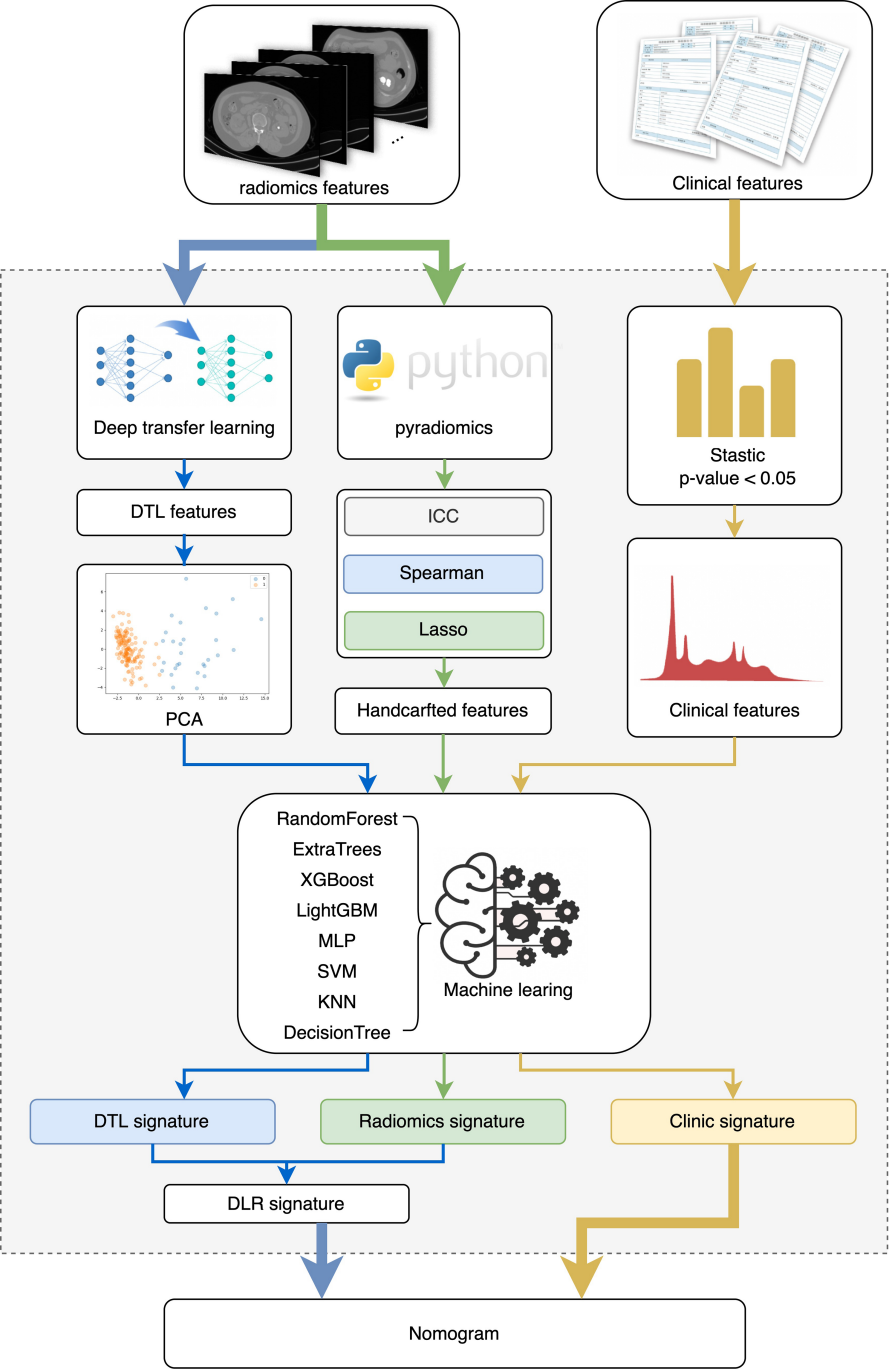
The study flowchart and the workflow of radionics.

This work presents a deep learning model for the prediction of conditions after PCNL in patients with renal staghorn stones. Hand-made characteristics were extracted from CT images by radiology. Depth characteristics are extracted from CT images by cropping the maximum area slice of the ROI. Depth characteristics are extracted from pre-trained resnet50 by transfer learning. The most robust non-redundant and predictive characteristics are selected using correlation filters and Lasso. Finally, a map of radiological characteristics and nominations was developed, and the content was shown in **Figure 2**.

## Methods

### Patients and population

This retrospective analysis obtained ethical approval and gave up informed consent requirement. Then we retrospectively included 175 patients who received PCNL from December 2017 to March 2022, including 112 patients from Shenzhen Hospital of Hong Kong University and 63 patients from Zhuzhou Hospital affiliated to Xiangya Medical College of Central South University. We actually have collected information on 175 patients (67 patients in the training set and 108 in the testing set) (**Table 1**).Relevant clinical data and characteristics of patients with renal calculi were extracted before surgery, including age, gender, degree of hydronephrosis, stone load and surgical experience, as well as other relevant data, such as surgeons’ experience. Postoperative follow-up procedures were carried out one month after the operation, and evaluation was made according to the CT or X-ray examination results (18).

**Table 1:** Baseline characteristics of the patients.

	Extra validation Cohort				Inner training cohort			
Name	Left2 ALL	Left2-train	Left2-test	P value	Left1-ALL	Left1-train	Left1-test	P value
Gender				0.5398				0.3723
Female	32 (0.5079)	18 (0.5455)	14 (0.4667)		50 (0.4464)	13 (0.3824)	37 (0.4744)	
Male	31 (0.4921)	15 (0.4545)	16 (0.5333)		62 (0.5536)	21 (0.6176)	41 (0.5256)	
Age (years)				0.6185				0.9274
<65	48 (0.7619)	26 (0.7879)	22 (0.7333)		83 (0.7411)	25 (0.7353)	58 (0.7436)	
≥65	15 (0.2381)	7 (0.2121)	8 (0.2667)		29 (0.2589)	9 (0.2647)	20 (0.2564)	
Stone laterality				0.9836				0.6094
Bilateral	3 (0.0476)	2 (0.0606)	1 (0.0333)		11 (0.0982)	6 (0.1765)	5 (0.0641)	
Left	34 (0.5397)	17 (0.5152)	17 (0.5667)		45 (0.4018)	10 (0.2941)	35 (0.4487)	
Right	26 (0.4127)	14 (0.4242)	12 (0.4000)		56 (0.5000)	18 (0.5294)	38 (0.4872)	
Pre-operative infection				0.0683				0.3176
No	11 (0.1746)	3 (0.0909)	8 (0.2667)		64 (0.5714)	17 (0.5000)	47 (0.6026)	
Yes	52 (0.8254)	30 (0.9091)	22 (0.7333)		48 (0.4286)	17 (0.5000)	31 (0.3974)	
Duration of surgery (minutes)				0.614				
<90	17 (0.2698)	8 (0.2424)	9 (0.3000)					
≥90	46 (0.7302)	25 (0.7576)	21 (0.7000)		112 (1.0000)	34 (1.0000)	78 (1.0000)	
Operator experience				0.6245				0.7026
Senior Physicians	23 (0.3651)	13 (0.3939)	10 (0.3333)		85 (0.7589)	25 (0.7353)	60 (0.7692)	
Junior Physicians	40 (0.6349)	20 (0.6061)	20 (0.6667)		27 (0.2411)	9 (0.2647)	18 (0.2308)	
Number of surgeries (total)				0.0526				0.0287
1	53 (0.8413)	25 (0.7576)	28 (0.9333)		33 (0.2946)	7 (0.2059)	26 (0.3333)	
2	9 (0.1429)	7 (0.2121)	2 (0.0667)		53 (0.4732)	13 (0.3824)	40 (0.5128)	
3	1 (0.0159)	1 (0.0303)	null		19 (0.1696)	12 (0.3529)	7 (0.0897)	
4	7 (0.0625)	2 (0.0588)	5 (0.0641)					
Number of channels				0.0351				0.3074
1	55 (0.8730)	26 (0.7879)	29 (0.9667)		71 (0.6339)	20 (0.5882)	51 (0.6538)	
2	7 (0.1111)	6 (0.1818)	1 (0.0333)		33 (0.2946)	10 (0.2941)	23 (0.2949)	
3	1 (0.0159)	1 (0.0303)	null		8 (0.0714)	4 (0.1176)	4 (0.0513)	
BMI				0.5292				0.3971
Normal	27 (0.4286)	14 (0.4242)	13 (0.4333)		54 (0.4821)	20 (0.5882)	34 (0.4359)	
Thin	5 (0.0794)	2 (0.0606)	3 (0.1000)		5 (0.0446)	2 (0.0588)	3 (0.0385)	
Overweight	25 (0.3968)	12 (0.3636)	13 (0.4333)		32 (0.2857)	3 (0.0882)	29 (0.3718)	
Obesity	6 (0.0952)	5 (0.1515)	1 (0.0333)		21 (0.1875)	9 (0.2647)	12 (0.1538)	
Pre-operative DJ stenting				0.6916				0.4154
No	49 (0.7778)	25 (0.7576)	24 (0.8000)		69 (0.6161)	19 (0.5588)	50 (0.6410)	
Yes	14 (0.2222)	8 (0.2424)	6 (0.2000)		43 (0.3839)	15 (0.4412)	28 (0.3590)	
Pre-operative ESWL				0.0673				0.089
No	57 (0.9048)	32 (0.9697)	25 (0.8333)		98 (0.8750)	27 (0.7941)	71 (0.9103)	
Yes	6 (0.0952)	1 (0.0303)	5 (0.1667)		14 (0.1250)	7 (0.2059)	7 (0.0897)	
History of previous multiple surgeries				0.3884				0.0274
No	45 (0.7143)	22 (0.6667)	23 (0.7667)		40 (0.3571)	7 (0.2059)	33 (0.4231)	
Yes	18 (0.2857)	11 (0.3333)	7 (0.2333)		72 (0.6429)	27 (0.7941)	45 (0.5769)	
Degree of hydronephrosis				0.9253				
Mild	40 (0.6349)	21 (0.6364)	19 (0.6333)		60 (0.5357)	14 (0.4118)	46 (0.5897)	
Moderate	13 (0.2063)	7 (0.2121)	6 (0.2000)		29 (0.2589)	7 (0.2059)	22 (0.2821)	
Severe	10 (0.1587)	5 (0.1515)	5 (0.1667)		15 (0.1339)	8 (0.2353)	7 (0.0897)	
None	7 (0.0625)	4 (0.1176)	3 (0.0385)					
Hypertension				0.1559				0.4576
No	45 (0.7143)	21 (0.6364)	24 (0.8000)		78 (0.6964)	22 (0.6471)	56 (0.7179)	
Yes	18 (0.2857)	12 (0.3636)	6 (0.2000)		34 (0.3036)	12 (0.3529)	22 (0.2821)	
Diabetes				0.4009				0.6775
No	53 (0.8413)	29 (0.8788)	24 (0.8000)		93 (0.8304)	29 (0.8529)	64 (0.8205)	
Yes	10 (0.1587)	4 (0.1212)	6 (0.2000)		19 (0.1696)	5 (0.1471)	14 (0.1795)	
Maximum diameter of the stone (mm)				0.3884				0.7235
<50	45 (0.7143)	22 (0.6667)	23 (0.7667)		88 (0.7857)	26 (0.7647)	62 (0.7949)	
≥50	18 (0.2857)	11 (0.3333)	7 (0.2333)		24 (0.2143)	8 (0.2353)	16 (0.2051)	
Maximum CT value of the stone (Hu)				0.9339				0.8779
<700	15 (0.2381)	8 (0.2424)	7 (0.2333)		14 (0.1250)	4 (0.1176)	10 (0.1282)	
≥700	48 (0.7619)	25 (0.7576)	23 (0.7667)		98 (0.8750)	30 (0.8824)	68 (0.8718)	
Volume of the stone (cm3)				0.9616				0.2702
<11.9	25 (0.3968)	13 (0.3939)	12 (0.4000)		68 (0.6071)	18 (0.5294)	50 (0.6410)	
≥11.9	38 (0.6032)	20 (0.6061)	18 (0.6000)		44 (0.3929)	16 (0.4706)	28 (0.3590)	

### Radionics procedure

Image acquisition. Baseline clinical, including age, sex and BMI, preoperative and intraoperative clinical characteristics were obtained from the medical records. The patient had a preoperative 64-row CT of the urinary tract (Discovery CT750 HD, GE. Healthcare, USA) perfected. The experienced radiologist also reviewed the pre-processed CT images and recorded data from the CT images. Any differences shall be resolved through consultation. Open source software 3D Slicer (www.slicer.org) is used to obtain CT images, segment regions of interest, and annotate ROI.

Image preprocessing: Heterogeneous voxel spacing is prevalent in multicenter medical volumes due to differences in scanners or acquisition protocols. Under different imaging, the range of pixel values in medical images varies widely from center to center. We use resampling methods to ensure intensity consistency across all data.

The manually extracted characteristics are divided into three main groups: geometric characteristics, intensity characteristics, and texture characteristics. Geometric characteristics describe the 3-D shape characteristics of the stone, and strength characteristics describe the first-order statistical analysis characteristics of the internal strength of the stone.

**Figure 3:**
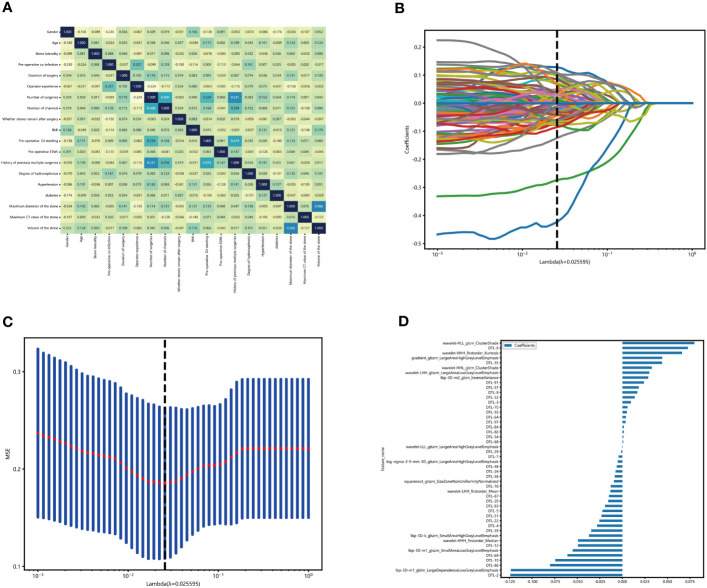
Construction of the clinical-radionics signatures. **(A)** We use the extra reels model to fit these clinical characteristics and establish the correlation map of clinical characteristics. The X and Y axes represent the characteristics used by the model. Each square code feature is related—the darker the color, the more correlation between artificial characteristics. In the figure, except for the diagonal, the highest score of feature correlation is 0.566(volume of the stone, maximum diameter of the stone). The higher the grid score, the weaker the fitting ability of the model fitting. **(B, C)** Sixty-three characteristics of non-zero coefficients were selected to establish the DTL-score + Rad-score with a least absolute shrinkage and selection operator (LASSO) logistic regression model (λ _= 0.18). **(D)** The histogram of the Rad-score.

The geometric characteristics describe the three-dimensional shape characteristics of the stone, and the intensity characteristics describe the first-order statistical analysis characteristics of the internal intensity of the stone. In contrast, the texture, second-order, and high-intensity spatial distribution characteristics are extracted using several different methods. The content of the hand-crafted characteristics can be found in **Figure 3**. All hand-crafted characteristics were extracted using the internal feature analysis procedure implemented in Pyradionics (http://pyradionics.readthedocs.io).

Feature selection: For characteristics with high repeatability, the Spearman rank correlation coefficient is used to calculate the correlation between characteristics. Only one of any two characteristics with a correlation coefficient greater than 0.9 was retained.

### Deep transfer learning procedure

Neural network characteristics are extracted from pre-trained CNN. In this study, resnet50 was chosen as the model and trained on the ILSVRC-2012 dataset. Select the slice with the largest ROI area to represent each patient. In addition, the conversion value was used to normalize the gray value to the range (– 1, 1). Next, each cropped sub-region image is adjusted to 224 × 224 using the nearest interpolation method. the resulting image can be used as model input. Next, principal component analysis (PCA) is used to reduce the dimension of depth migration features to ensure the balance between features. We reduce the dimensionality of deep learning to 100 dimensions to improve the model’s generalization ability and reduce the risk of overfitting.

### Construction of radionics signature and deep transfer learning signature

After PCA compresses deep learning characteristics, all radiological characteristics are normalized by the z-score normalization method. Then, applying the minor absolute shrinkage and selection operator logistic regression algorithm to select features with non-zero coefficients from the training cohort through 10-fold cross-validation for penalty parameter adjustment. A radiographic feature is generated by combining the selected characteristics and weighting them by their respective coefficients. Next, the mean and variance (STD) were calculated for each feature column. Each column of characteristics is subtracted from the mean, divided by the variance, and then transformed into a standard normal distribution. Continue to use the minimum absolute shrinkage and selection lasso to filter out characteristics with non-zero coefficients, select and reduce the dimensionality of the fused characteristics, and find the best subset of fused characteristics. Finally, 76-dimensional deep migration learning characteristics are obtained.

### DTL+Rad-signature

Based on selected radiological signatures, and 100 compressed deep migratory learning signatures, we aim to construct a deep learning radiological signature. We follow the same pipeline as for the radiology signature or the deep migration learning signature. After Lasso feature screening, we feed the final characteristics into a machine learning model for risk model construction to obtain the final DTL+Rad signature. See also Deep Learning Radiology Signature (DLR).

### Validation of radionics models

External and internal validation was aim to validate the accuracy of the radiology model. There are 67 patients in the training set and 108 in the testing set. Calculating the risk score for patients, according to the risk score of patients. In addition, the model proposed in this paper is also evaluated for discrimination and calibration.

### Clinical utility of radionics models

The clinical utility of the prediction model is determined according to obtaining the data income under different threshold. Then, all the selected patients were analyzed by operating parameters to compare whether the radiology model has good efficacy and clinical application in predicting the one-time stone removal rate after PCNL in patients with renal stones.

### Statistical analysis

Using statsmodels python package version 0.13.2 to conduct Statistical analyses. The Kolmogorov-Smirnov test assessed the normality of all continuous variables. Logistic regression with univariate and multivariate statistical analyses was conducted to charify critical paremeters.

## Results

### Clinical characteristics

**Table 1** summarizes the result of the training and validation group. About 16.0% of patients (67 of 175) had residual stones after undergoing a PCNL procedure. In the training set, 31% of patients had residual stones after undergoing another PCNL procedure. The incidence of residual stones was 31%, and the rate of one-time stone removal was 69%. In contrast, the incidence of residual stones after PCNL was 53% in the external validation group. **Table 1** shown the stone composition parameters of all enrolled patients. By analyzing the covariance of each characteristic, we found that the clinical characteristics showed less correlation (**Figure 3A**).

### Feature selection, radionics signature construction, and validation

According to the CT images, 1734 radiological parameters were obtained. The expected reproducibility of inter-observer feature extraction was achieved because the edges of urinary stones were evident in the CT images. We used the extracted 1734 radiological characteristics, 2048 deep learning characteristics based on migration learning, and early fusion techniques to fuse these characteristics to obtain 3782 deep learning radiological characteristics. We performed lasso analysis, correlation coefficient screening, and PCA dimensionality reduction for these characteristics. We filtered out 48 characteristics with non-zero coefficients, including 14 radiology characteristics and 34 migration learning characteristics. Then, we built DLR characteristics. (**Figures 3B, C**). These radiological characteristics and their corresponding characteristics are as follows (**Figure 3D**).

### Prediction models

We constructed radiology-clinical correlation models by machine learning and transfer learning algorithms. Then, we compared the different models in the training and validation group and discovered that DTL+Rad signature performed the best predictions. The AUC values of DTL+Rad-Signature were 0.871 and 0.744, the sensitivities were 0.634 and 0.733, and the specificities were 0.806 and 0.935 in the external and training validation groups (**Table 2**). It indicated that DTL+Rad-signature has good predictive efficacy in predicting stone clearance after PCNL. The DTL+Rad signature was calculated as follows:

**Table 2:** Comparison of Signatures: We used ROC to compare the effectiveness of different signatures in the final prediction results, as follows:.

Signature	Accuracy	A.U.C.	95% CI	Sensitivity	Specificity	Threshold	Task
Clinic-Signature	0.7321	0.8839	0.8087 - 0.9591	1.0000	0.6765	0.6206	Train
Clinic-Signature	0.4761	0.6010	0.4589 - 0.7431	0.2667	0.9394	0.8123	Test
Rad-Signature	0.7560	0.8190	0.7372 - 0.9008	0.6667	0.8125	0.7000	Train
Rad-Signature	0.5873	0.7051	0.5748 - 0.8353	0.6333	0.7667	0.9000	Test
DTL+Rad-Signature	0.7157	0.8707	0.7999 - 0.9416	0.6338	0.9355	0.7115	Train
DTL+Rad-Signature	0.4762	0.7440	0.6166 - 0.8713	0.7333	0.8064	0.7067	Test
Nomogram	0.7460	0.7768	0.6616 - 0.8919	0.7333	0.7576	0.4870	Test

DTL+Radionics score = 0.6534968892241029 -0.124189 * DTL-2 + 0.009462 * DTL-3 -0.028079 * DTL-4 -0.022059 * DTL-5 + 0.072624 * DTL-6 -0.004288 * DTL-7 + 0.016654 * DTL-9 -0.074388 * DTL-10 -0.049319 * DTL-12 -0.015766 * DTL-20 -0.025414 * DTL-22 -0.007556 * DTL-24 -0.001361 * DTL-29 + 0.013734 * DTL-32 + 0.043993 * DTL-35 -0.008485 * DTL-36 -0.034550 * DTL-39 -0.006323 * DTL-48 -0.023089 * DTL-51 + 0.001158 * DTL-54 + 0.005054 * DTL-55 + 0.018219 * DTL-57 + 0.003968 * DTL-64 + 0.001042 * DTL-68 -0.060856 * DTL-69 + 0.005685 * DTL-70 -0.012981 * DTL-76 + 0.001676 * DTL-80 + 0.001739 * DTL-84 -0.080262 * DTL-86 -0.014884 * DTL-87 + 0.023936 * DTL-91 -0.019297 * DTL-93 + 0.003644 * DTL-97 + 0.044201 * gradient_glszm_LargeAreaHighGrayLevelEmphasis -0.037085 * lbp-3D-k_glszm_SmallAreaHighGrayLevelEmphasis -0.123581 * lbp-3D-m1_gldm_LargeDependenceLowGrayLevelEmphasis -0.055118 * lbp-3D-m1_glszm_SmallAreaLowGrayLevelEmphasis +0.028711 * lbp-3D-m2_glcm_InverseVariance -0.005210 * log-sigma-2-0-mm-3D_glszm_LargeAreaHighGrayLevelEmphasis -0.009336 * squareroot_glszm_SizeZoneNonUniformityNormalized +0.066162 * wavelet-HHH_firstorder_Kurtosis -0.049268 * wavelet-HHH_firstorder_Median +0.032635 * wavelet-HHL_glcm_ClusterShade +0.079756 * wavelet-HLL_glcm_ClusterShade -0.013344 * wavelet-LHH_firstorder_Mean +0.029856 * wavelet-LHH_glszm_LargeAreaLowGrayLevelEmphasis -0.000432 * wavelet-LLL_glszm_LargeAreaHighGrayLevelEmphasis

**Figure 4:**
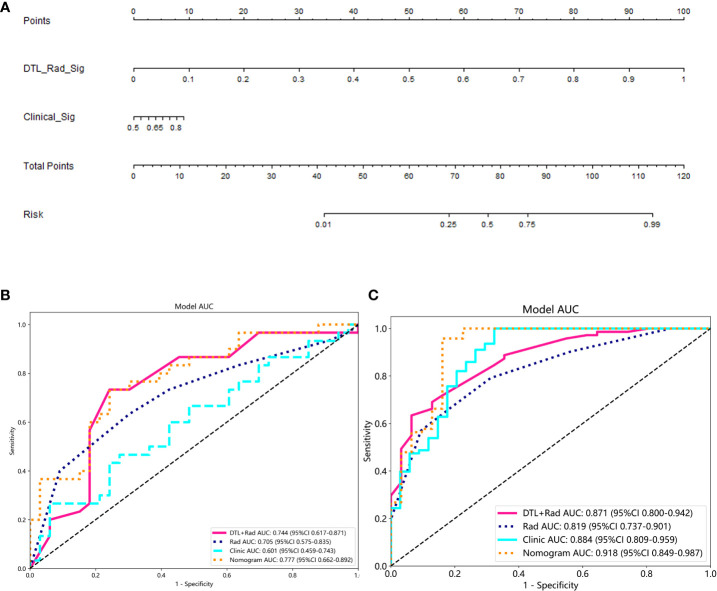
Performance of the clinical-radionics model. **(A)** Nomogram developed based on the clinical-radionics model. **(B, C)** Receiver operating characteristic (ROC) curves of the radionics model in the training and external validation groups for the radionics model, respectively.

The probability of one-time clearance of renal cast stones treated with PCNL was obtained by 1/[1 þ exp (risk score)]. A nomogram via the radiological model is developed to offer a convenient method is shown as the radiological model (**Figure 4A**, **Table 2**).

### Radionics model validation

**Figure 5:**
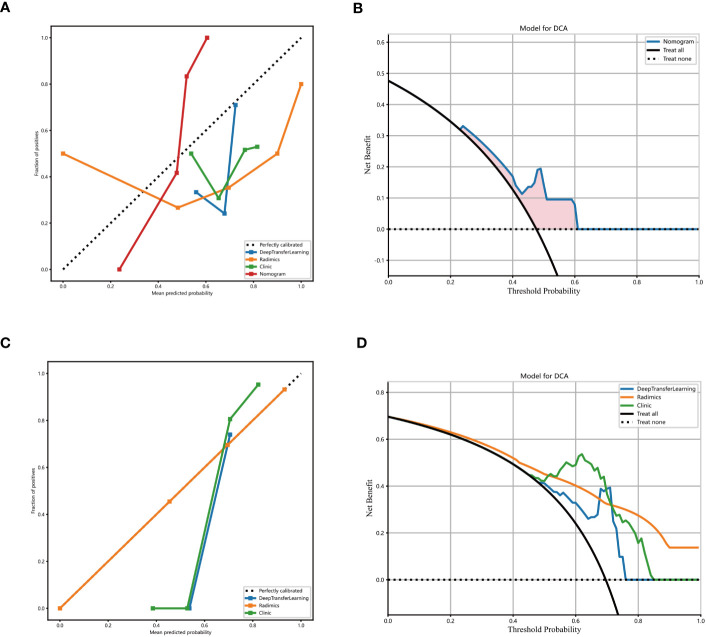
Decision curve analysis of the radionics model. The blue line represents the DTL+ radionics model. The black line represents the assumption that all patients have no stones remaining after PCNL in patients with renal cast stones. The yellow line represents the radionics model. The green line represents the clinical-only model **(A)** training set. **(B)** External validation set. **(C, D)** Calibration curves of the DTL+ radionics model. x- and y-axes show the predicted and actual probability of complete clearance of one-time stones after PCNL in patients with renal cast stones, respectively. The calibration curve depicts the calibration of the model, i.e, the agreement between the predicted and actual probabilities. Agreement between predicted and observed probabilities.

The proposed model showed good discrimination ability in the training group, with the AUC of 0.871 (95% CI, 0.800-0.942) (**Figure 4B**). **Figure 4C** shows the better accuracy of the model. Unexpectedly, the validation group obtained great result with an AUC of 0.744 (95% CI, 0.617-0.871) (**Figure 4C**). Encouragingly, its calibration curve showed an excellent radiological model calibration (**Figures 5A, C**). The above results demonstrate the consistency, generalization ability and good fit of the DTL+Radionics signature.

### Clinical usefulness of the radionics model

The prediction performance of DLR combined with radiological signatures and deep migration learning signatures was improved based on comparing each individual signature. A nomogram for visual recognition was built using the predictive model of DLR combined with clinical signatures (**Figure 4A**,**Table 2**). We also validated the nomogram’s results and found that integrating deep learning radiology signature and clinical signature gave better results (AUC=0.777) than dtl+rad Signature (AUC=0.744)(**Table 2**). Decision curve analysis showed promising efficacy in using the radiological model to predict one-time stone clearance after receiving PCNL for renal stones (**Figures 5B, D**). The above evidence hypothesizes that the model yields good predictive power in predicting postoperative one-time stone clearance treated with PCNL direction.

## Discussions

Staghorn stones are a unique and complex subtype of kidney stones. PCNL is the basic guidelines for the therapy of staghorn stones. Despite the continuous development of PCNL techniques, staghorn stones remain a challenge for doctors because of higher perioperative complication rates than non-staghorn stones. Common intraoperative complications include hemorrhage, renal collecting system injury, visceral organ injury, pulmonary complications, extrarenal stone displacement, nephrostomy tube dislocation, and complications of venous thromboembolism (19). Therefore, it is crucial to accurately assess the outcome of PCNL surgery in advance and select the right patients. Precision medicine promotes in-depth research on targeted disease treatment, and the extraction of individual disease phenotypes is a prerequisite for the development of precision medicine. Imaging histology uses radiological imaging techniques to extract various radiological markers with the help of specific algorithms. In other words, imaging histology is applied by converting characteristics of MRI, CT, and other images into quantifiable data through computer-aided diagnostic techniques. With this technique, abstract image characteristics can be expressed indirectly in terms of concrete objective numbers, and the extracted data are analyzed to build predictive models. Imaging histology has been widely used to identify malignancy of urological tumors, postoperative survival prediction, and preoperative lymph node metastasis (20–23). With the increasing popularity of imaging histology, it is now used to diagnose kidney stones (24–26). For example, the prognosis and prevention of kidney stones can be guided depended on analyzing composition of the patient’s urinary tract stones. The timing and extent of laser or ultrasound lithotripsy and the parameters of the stones are considered essential factors in determining the surgical options for urinary stones (27, 28). Antler-shaped stones require a longer operative time. They are more likely to remobilize during surgery, and movement may result in stone retention.

However, a comprehensive analysis of stones before surgery remains a significant challenge. Firstly, the current analysis method can only rely on postoperative or intraoperative detection. Neither postoperative nor intraoperative testing is feasible for preoperative evaluation. Then, simple measurement of stone density using HU (units of CT) is not advisable because of the heterogeneous and complex situation within the renal deuterostomes. This may explain why many studies have not yet included stone density in their assessment scoring systems (29, 30).

This article establishes an easy-to-apply and standardized preoperative assessment tool for PCNL that will aid in clinical decision-making, evaluation of surgical outcomes, and academic research in patients with kidney stones (31, 32).

These tools allow analysis of the safety and reliability of kidney stone surgery and clinical studies (33). The most common, validated analysis systems predict SFR and complications after PCNL (34). The GSS consists of four grades due to the stone structure and the patient’s condition. CROES is based on global data and is highly versatile. While the STONE divides patients into three groups, which is helply to make decisions (35, 36). Although these analysis systems include different parameters. Stone location, number of stones, and staghorn stones are critical variables in all analysis systems (35). However, no comprehensive analysis system for predicting the prognosis of patients after PCNL surgery, and there is an apparent conflict between different authors on the analysis system for the prognostic prediction function. Several experts reported that all anslysis systems were valid and so as the SFR when estimated and compared in 246 post-PCNL patients (37). Tailly reported similar result of the three analysis systems for SFS by comparing the three analysis systems in 586 post-PCNL patients. However, there was no association between the three analysis systems and complications (13).

The present study identified a set of 76 strongly correlated features as an independent factor for stone clearance rate (SFR) in patients with PCNL. This multi-feature-based radiological signature successfully divided patients into a clearance group and a residual group in the validation dataset. Other studies on extracorporeal shock wave lithotripsy (SWL) have found that quantitative analysis of enhanced CT can ameliorate medical decision-making with ESWL (38). Similarly, some studies suggest that building predictive models using radiology or machine learning may help improve preoperative outcome prediction in PCNL (16, 39, 40). A single strong risk indicator (radiological features) may not be sufficient to assess the postoperative situation of patients. Hence, a deep learning-based clinical-radiological model is conducted by combining the clinical characteristics of patients. It is a combination of radiological characteristics and potentially valuable clinical characteristics. To validate the value of different data and algorithms for the final stoneless rate goal, we constructed different signatures using the Extra-Trees algorithm for three different data: clinical data, imaging characteristics, and deep migration learning characteristics + imaging characteristics to investigate the impact of additional data and methods. This study found that nomograms that combine deep learning, imaging, and clinical characteristics yield better results. In addition, decision curve analysis showed that the benefit of deep learning-based clinical-radiological nomograms was more significant. Decision curve analysis shows that clinical-radiological nomograms are more beneficial than all-or-none treatment options.

In terms of clinical predictors, the evaluation of our findings is consistent with the current mainstream studies. These mainstream studies also agree that kidney stone location and volume, degree of hydronephrosis are important parameters for SFR after PCNL (29, 34, 41). Domenico Viola and Silvia Proietti reported that the size of the stone influenced the success of PCNL, and our study showed similar results (42, 43). As the stone size increases, it takes longer and the stone fragments migrate more easily. It also increases the chance of intraoperative bleeding, negatively affects the surgical field, and results in residual stones after surgery. In addition, our findings are consistent with Chen Ke’s research. We believe that the degree of hydronephrosis correlates with SFR due to renal pelvis and calyx, complicating lithotripsy and increasing residual stones after surgery (44). In addition, an experienced surgeon is a critical factor in the outcome of PCNL, mainly in terms of safety (43, 45). In previous study, surgeons had to perform approximately 24 PCNLs to achieve good proficiency, operate on 60 patients to achieve standard PCNL competence, and perform more than 100 PCNL procedures to achieve excellence (32). Likewise, it was found that operators with more than 100 surgical experience had a high level of familiarity and could effectively manage complications. In addition, skilled use of ureteroscopes and assistive devices during surgery can prevent some life-threatening complications.

Although Bozzini reported that FURS provided better SFR and lower retreatment rates among the various treatments for kidney stones (46), this study showed that FURS had better results than PCNL. However, De et al. (47)showed that the stoneless rate was strong correlation with PCNL than with fURS. In a preliminary search of 553 articles, Amelia Pietropaol compared different approaches to kidney stone surgery, including PCNL, URS, and SWL, with final SFRs of 67%-97.7%, 43%-100%, and 73%-80% (48). Our study’s overall SFR for PCNL was approximately 72%, consistent with other studies (range 67% to 97%). Several investigators have studied specific factors that influence SFR, such as the pelvic floor angle (IPA). They reported that IPA and other pelvic anatomy-related parameters were associated with lower SFR (49). However, the result shown that it was impossible to demonstrate the effect of pelvic collection system anatomy on stone fragment removal from the hypocalyx, and the effect of hypocalyx anatomy on stoneless rates remains controversial (50). In addition, the measurement of IPA is controversial: urologists rely on intravenous urography (IVU) or contrast-enhanced CT (CCT) to measure IPA. However, patients generally do not receive either of these tests. In addition, patients who are allergic to contrast agents or have moderate to severe renal insufficiency are not candidates for this test. Finally, due to the perfusion of water during the procedure, the renal pelvis and calyces are distended and preoperative measurements cannot be an accurate predictor during this procedure.

In this study, imaging histological characteristics variables were used for analysis in combination with pre-clinical treatment factors. This is a non-invasive and reproducible technique that is not influenced by the individual patient. In addition, using computer-aided diagnostic methods to extract and analyze information from a patient’s image picture, construct predictive models, and plot histograms can help clinicians read image information that cannot be identified by the naked eye. Thus, imaging histology has good potential for clinical application due to its high utility as an important tool for precision medicine. But this paper still has some deficiencies. First, this article is a review of previous research, and may miss some duplicative results. Furthermore, our proposed model is built on one dataset and validated on two other datasets, which may not be convincing enough. Therefore, more verification work will be carried out in the future.

## Conclusions

We found that a prediction model combining deep migration learning, imaging characteristics and clinical characteristics can be used as an comprehensive method for stone removal rate in PCNL surgery for clinical decision making in patients with renal staghorn stones. When choosing a PCNL treatment strategy, criteria like a smaller volume of stones, a lesser degree of hydronephrosis, and an experienced surgeon are more likely to be successful.

## Data availability statement

The original contributions presented in the study are included in the article/supplementary material. Further inquiries can be directed to the corresponding author.

## Ethics statement

Written informed consent was obtained from the individual(s) for the publication of any potentially identifiable images or data included in this article.

## Author contributions

XW and JH designed the manuscript. JH and XW wrote the main manuscript. GQW, RJ, GQ, WC, and LX complete ROI segmentation. XX, GGW, TL, and WH completed the data collecting and analysis. All authors contributed to the article and approved the submitted version.
